# Enhance the Pyroelectricity of Polyvinylidene Fluoride by Graphene-Oxide Doping

**DOI:** 10.3390/s140406877

**Published:** 2014-04-16

**Authors:** Yuh-Chung Hu, Wei-Li Hsu, Yi-Ta Wang, Cheng-Tao Ho, Pei-Zen Chang

**Affiliations:** 1 Department of Mechanical and Electromechanical Engineering, National ILan University, No. 1, Sec. 1, Shenlung Road, ILan 26041, Taiwan; E-Mail: ytwang@niu.edu.tw; 2 School and Graduate Institute of Physical Therapy, National Taiwan University, No. 1, Sec. 4, Roosevelt Road, Taipei 10617, Taiwan; E-Mail: wlhsu@ntu.edu.tw; 3 Institute of Applied Mechanics, National Taiwan University, No. 1, Sec. 4, Roosevelt Road, Taipei 10617, Taiwan; E-Mails: jdh78517@gmail.com (C.-T.H.); changpz@ntumems.net (P.-Z.C.)

**Keywords:** flexible electronics, graphene-oxide, PVDF, pyroelectric, sol-gel 1. Introduction

## Abstract

The high quality properties and benefits of graphene-oxide have generated an active area of research where many investigations have shown potential applications in various technological fields. This paper proposes a methodology for enhancing the pyro-electricity of PVDF by graphene-oxide doping. The PVDF film with graphene-oxide is prepared by the sol-gel method. Firstly, PVDF and graphene-oxide powders are dispersed into dimethylformamide as solvent to form a sol solution. Secondly, the sol solution is deposited on a flexible ITO/PET substrate by spin-coating. Thirdly, the particles in the sol solution are polymerized through baking off the solvent to produce a gel in a state of a continuous network of PVDF and graphene-oxide. The final annealing process pyrolyzes the gel and form a β-phase PVDF film with graphene-oxide doping. A complete study on the process of the graphene oxide doping of PVDF is accomplished. Some key points about the process are addressed based on experiments. The solutions to some key issues are found in this work, such as the porosity of film, the annealing temperature limitation by the use of flexible PET substrate, and the concentrations of PVDF and graphene-oxide.

Stimuli-responsive polymers have received considerable attention due to their singular electromechanical properties, which make them materials of choice for numerous applications. Polyvinylidene fluoride (PVDF) is a polymeric piezoelectric and pyroelectric material and its copolymers with trifluoroethylene (TrFE) or tetrafluoroethylene (TFE) are well known typical ferro-electric polymers [1,2]. PVDF has several crystal modifications that depend on the sample preparation conditions among which the α-phase and β-phase (polar phase) are the most relevant for practical piezoelectric and pyroelectric applications [3–6]. Among these polymorphs, β-phase crystals, consisting of parallel packing of all *trans* chains in an orthorhombic unit cell, exhibit large spontaneous polarization [3–6] and a high modulus because the β-phase produces a net dipole moment. Mechanical stretching [1] is generally utilized in order to obtain highly orientated β-phases, but this type of process is not appropriate for thin films. In another way, β-phases can be prepared by the cold drawing at a low temperature of melt-quenched films consisting of α-phase, but drawing at higher temperatures gives a mixture of α- and β-phases [1]. A cold-drawn β-phase has lower crystallinity and its electrical properties are lower than those of the copolymers of vinylidene fluoride (VDF) and TrFE with high VDF contents, which commonly crystallize in the β-phase, with higher crystallinity approaching 100% [1]. Recently, ferroelectric PVDF films have started to be employed as tactile sensors in robotic applications [7–9]. Although many publications have reported the effects of annealing on the structure and properties of VDF/TrFE copolymers [10,11], only a few such studies concerning β-phase PVDF have been published. A number of papers have also demonstrated that the addition of external nucleating agents, such as carbon nanotubes [12], modified graphene nanosheets [13,14] or clay particles [15], can induce β-phase formation in PVDF nanocomposites obtained by solution casting. In those cases, however, the formation of piezoelectric β-phase is achieved only after special modifications of the fillers' surfaces to improve their capability to adsorb PVDF chains. The high adhesion found at the PVDF-filler interface causes a transformation of the α-phase *trans-gauche* (TG) conformation into the *trans-trans* (TT) conformation characteristic of the β-phase. Many reports have shown that incorporating a carbonyl group-containing filler into PVDF results in their homogeneous dispersion because of the strong and specific interactions between the fluorine group of PVDF and the carbonyl groups at the filler surface [12,13]. This in turn results in the formation of a β-phase structure as well as enhancements of the electrical, thermal and mechanical properties of such nanocomposites. Graphene-oxide (GO) can also be used as a nucleating agent for PVDF to produce high performance nanocomposite materials. GO is a single layer of graphite oxide obtained through a process of oxidation of natural graphite and essentially graphene sheets on which oxygen-containing functional groups are thought to be present in the form of carboxyl, hydroxyl and epoxy groups [16]. These oxygen-containing groups give hydrophilic characteristics to the GO layers and facilitate the intercalation of water molecules in the interlayer galleries. The desirable properties and benefits of GO have generated an active area of research where many investigations have shown potential applications in various technological fields. Because of their very hydrophilic character, an aqueous colloidal suspension of GO nanosheets can easily be obtained by complete exfoliation of bulk graphite oxide via simple sonication in water [16]. This paper proposes a methodology for enhancing the pyroelectricity of PVDF by GO doping. The GO-doped PVDF film is prepared by a sol-gel method. Firstly, PVDF and GO powders are dispersed into dimethylformamide (DMF) as solvent to form a sol solution. Secondly, the sol solution is deposited on a flexible ITO/PET substrate by spin-coating. Thirdly, the particles in the sol solution are polymerized by baking off the solvent to produce a gel in the form of a continuous network of PVDF and GO units. The final annealing process pyrolyzes the gel and forms a β-phase PVDF film with GO doping.

## 2. Methodology

The following Sections present the methodology for enhancing the pyroelectricity of PVDF by GO doping. The issues to be addressed and the equipment for each process are listed in Table 1.

### 2.1. Preparation of PVDF Sol Solution

The PVDF sol solution is formed by dispersing PVDF powder into a solvent. PVDF is an amorphous polymer, which exits in several forms: α-, β-, and γ-phases, depending on whether the chain conformations present *trans* (T) or *gauche* (G) linkages. β-Phase PVDF possesses pyroelectric properties while α-phase does not. The rate of evaporation of the solvent in the baking process directly influences the crystal structure of PVDF. Low evaporation rates result in β-phase PVDF while high evaporation rates result in α-phase, and medium evaporation rates will result in α- and β-phase simultaneously. However, the evaporation rate of the solvent also influences the porosity of the PVDF film. Lower evaporation rates result in higher porosity film which is a drawback for the application of PVDF film. Therefore, there is a trade-off between the β-phase crystallization and film porosity of PVDF with the selection of the solvent. The common solvents used in preparing PVDF sol solution are acetone, DMF, N-methyl-2-pyrrolidone (NMP), and hexamethylphosphoramide (HMPA) [17]. To reduce the porosity of PVDF film, we adopted DMF because it has the second highest evaporation rate among the aforesaid four solvents. A high evaporation rate is unfavorable for the β-phase crystallization of PVDF, but one can modify the β-phase crystallization via an annealing process. Three concentrations of the solution of PVDF in DMF are prepared in this process, whose weight percentages of PVDF are 5%, 10%, and 15% respectively. The PVDF powder (purity > 99%) in this work was provided by Sigma-Aldrich (St. Louis, MO, USA) while the DMF (purity > 99%) was obtained from Acros-Organics (Geel, Belgium). PVDF powder is dispersed in DMF and stirred for 4 h at 60 °C and then the solution is put into an ultrasonic cleaner bath and and shaken for 3 h till the PVDF powder was completely dispersed.

### 2.2. Spin-Coating of the PVDF Film

The PVDF film is deposited on a flexible ITO/PET substrate by spin-coating. Before spin-coating, the substrate must be cleaned carefully. The cleaning process involves immersing the substrate in an ultrasonic bath filled with acetone for 10 min, then with ethanol for 10 min, and then with de-ionized water for 10 min. Then, the PVDF sol solution is spin-coated on the substrate at a speed of 400 rpm for 10 s followed by 1,000 rpm for 10 s. Finally the solvent is baked off on a hot plate at 60 °C for 30 min. The film properties, including adhesion, porosity and thickness, are dependent on the PVDF concentration, spin-coating recipe (spinning speed and time), and solvent-baking (temperature and environment) step. As mentioned in Section 2.1, three kinds of PVDF concentrations are prepared, namely 5 wt%, 10 wt%, and 15 wt%, respectively. The best PVDF concentration, spin-coating recipe, and solvent-baking temperature and environment were determined by observing the film quality through an optical microscope.

### 2.3. Annealing of the PVDF Film

As mentioned in Section 2.1, DMF is unfavorable for the β-phase crystallization of PVDF though favorable for reducing film porosity. Therefore, after the spin-coating process, an annealing process is needed to modify the β-phase crystallization of PVDF. After finding a better PVDF concentration for film spin-coating (Section 2.2), the best annealing temperature for the β-phase crystallization of PVDF must be determined. The crystallization, infrared spectrum of absorption, and ultraviolet-visible spectrum of absorption of PVDF film are studied by X-ray diffraction (XRD), Fourier transform infrared spectroscopy (FTIR), and ultraviolet-visible spectrometry (UV-Vis) respectively. The remnant polarization of PVDF film is studied by observing the electrical hysteresis loop of the PVDF film.

### 2.4. Graphene Oxide Doping of the PVDF

**Figure 1.**
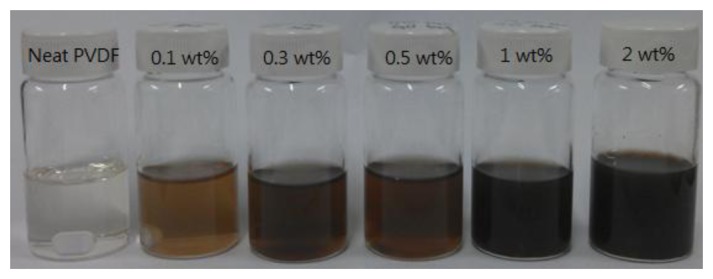
The solutions of GO-doped PVDF in DMF.

By the processes described in Sections 2.1–2.3, one can determine the best film-deposition and crystallization conditions for the PVDF film. After that, the GO doping of the PVDF is performed. The single-layer graphene oxide powder is produced by Legend Star International (Taipei, Taiwan). Firstly, GO powder was dispersed in DMF and stirred for 20 h at 60 °C till the GO powder was completely dispersed. Secondly, PVDF powder was added into the solution and stirred for 4 h at 60 °C till the PVDF powder was dispersed completely. Finally, the solution was placed in an ultrasonic cleaner and oscillated for 3 h till completely dispersed. Five different concentrations of GO-doping were prepared; the recipes and the resulting solutions are shown in Table 2 and Figure 1, respectively. Like in Sections 2.2 and 2.3, the GO-doped PVDF film is deposited on a flexible ITO/PET substrate by spin-coating and the β-phase crystallization modified by annealing.

### 2.5. Pyroelectricity Measurement of GO-Doped PVDF Film

A pyroelectric material will output a pyroelectric current *I_p_* when subject to temperature variation, and the relationship between the pyroelectric current and temperature variation is given by:

(1) $$I_{p}=A\cdot \gamma \cdot \frac{dT}{dt}$$

where *A* and *γ* are the electrode area and pyroelectric coefficient, respectively, and *dT*/*dt* is the temperature variation rate. A 10TEC-150 thermoelectric cooler produced by Unice E-Q Service Corporation (Taoyuan, Taiwan) is adopted as a heat source for measuring the pyroelectricity of the GO-doped PVDF film. The GO-doped PVDF film in placed on the thermoelectric cooler. One electrode of the GO-doped PVDF film is connected to the ground and the other one to a current-to-voltage converter which converted the tiny current (several nA) put out by the GO-doped PVDF film to voltage. The output voltage of the converter is measured by a multimeter. By Ohm's law, the tiny pyroelectric current put out by the GO-doped PVDF film can be deduced from the output voltage of the converter. The temperature variation range is 30 to 150 °C and the heating rate is 60 °C/min.

## 3. Results and Discussion

### 3.1. The Spin-Coating of PVDF Film

**Figure 2.**
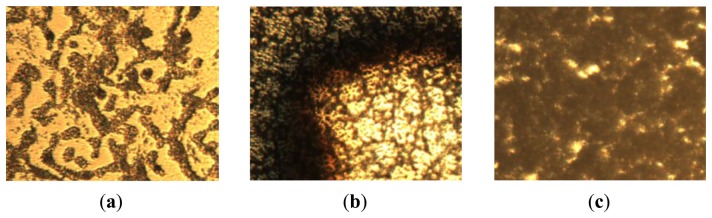
Optical microscope pictures (400×) of PVDF films after solvent-baking in the atmosphere. The weight percentages of PVDF are (**a**) 5 wt%; (**b**) 10 wt%; (**c**) 15 wt%.

**Figure 3.**
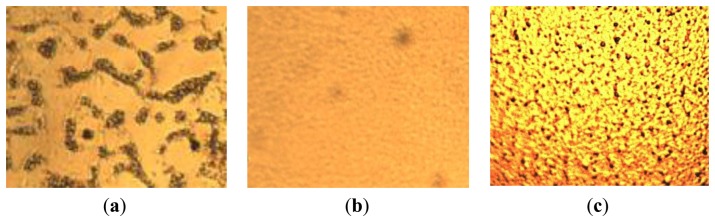
Optical microscope pictures (400×) of PVDF films after solvent-baking under vacuum (under 10 mTorr). The weight percentages of PVDF are: (**a**) 5 wt%; (**b**) 10 wt%; (**c**) 15 wt%.

The main disadvantage of the sol-gel method is the film porosity. As shown in Figure 2, the optical microscope pictures (400×) of PVDF films produced by solvent-baking in the atmosphere show many void fractions (the dark areas), which are formed during the solvent baking process. The porosity is harmful to film formation and increases the film roughness. No matter what the PVDF concentration or the spinning speed are, the porosity cannot be improved. Therefore, we tried to bake out the solvent under vacuum (under 10 mTorr) and thus found that the porosity can effectively be eliminated (Figure 3). For the case of 10 wt% PVDF, there is almost no porosity after solvent-baking under vacuum, while small void fractions are still seen in the case of 5 wt% and 15 wt% PVDF. This phenomenon is attributed to the fact that for the case of 5 wt% PVDF, parts of the solvent evaporate during the spin-coating step, while for the case of 15 wt%, the high concentration of PVDF prevents the venting of the air bubbles in the solvent during the solvent baking under vacuum. In summary, the film porosity can be effectively eliminated by s solvent-baking step performed under vacuum accompanied by the best PVDF concentration. Therefore, we determined the 10 wt% PVDF to be the best and adopted solvent-baking under vacuum in this work.

### 3.2. The Crystallization of PVDF

**Figure 4.**
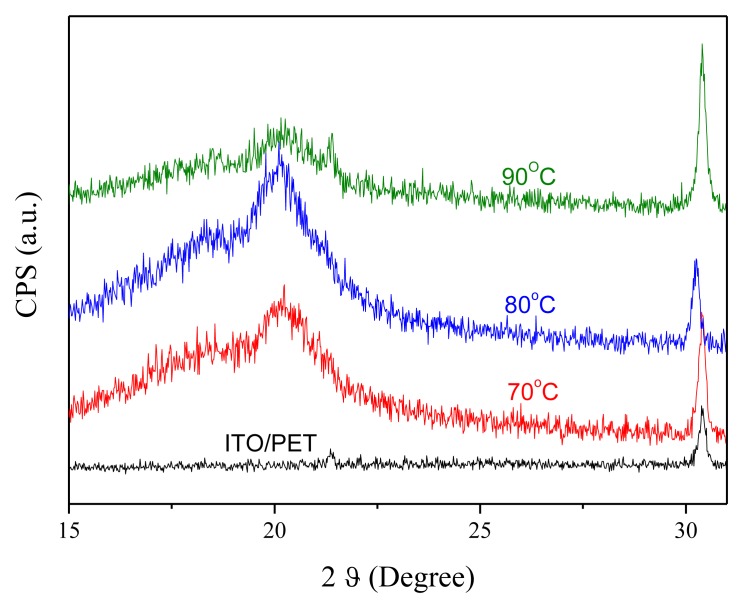
The XRD analysis of 10 wt% PVDF film under different annealing temperatures.

**Figure 5.**
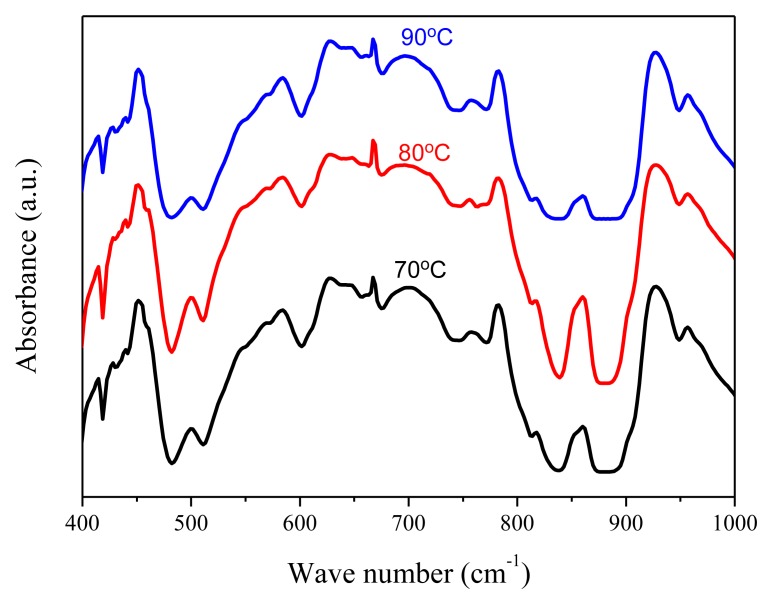
The FTIR analysis of 10 wt% PVDF film under different annealing temperatures.

**Figure 6.**
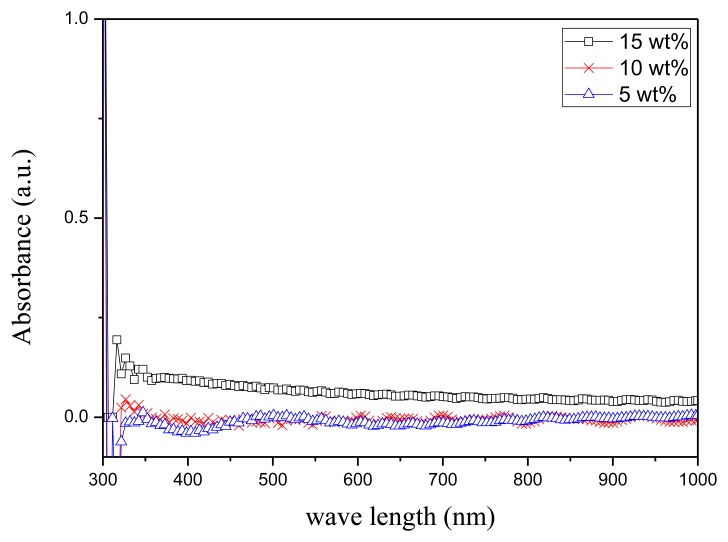
The UV-Vis analysis of PVDF film with different PVDF concentrations.

**Figure 7.**
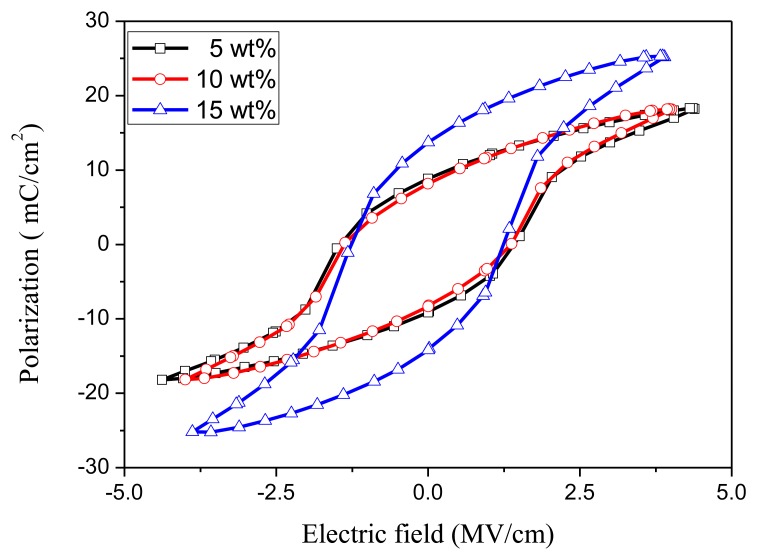
The remnant polarization of PVDF films with different PVDF concentrations, which are annealed at 80 °C for 2 h.

**Figure 8.**
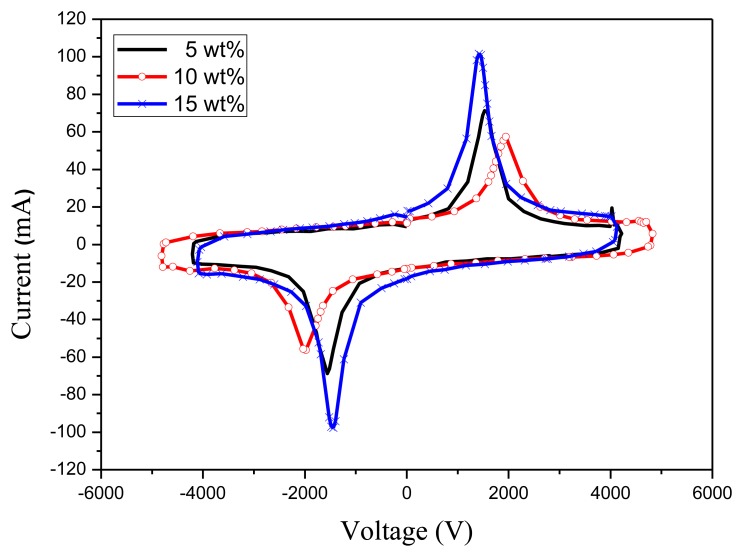
The C-V curves of PVDF films with different PVDF concentrations, which are annealed at 80 °C for 2 h.

An annealing process is required to modify the β-phase crystallization of PVDF after the film deposition process. Figure 4 shows the XRD analysis of the 10 wt% PVDF film under the annealing temperatures of 70 °C, 80 °C, and 90 °C for 2 h. The crystallization of PVDF is mainly β-phase (2*θ* = 20.7°) and α-phase (2*θ* = 17.9°) while the peak at 2*θ* = 30.4° is the ITO/PET substrate. One can find that the best annealing temperature for β-phase crystallization is 80 °C. Figure 5 shows the FTIR analysis of the 10 wt% PVDF film after annealing at temperatures of 70 °C, 80 °C, and 90 °C for 2 h. The peaks at the wave numbers 490, 511, 761 and 983 cm^−1^ correspond to the α-phase while those of 440, 477 and 840 cm^−1^ correspond to the β-phase [18–20]. Focusing on the peak at 840 cm^−1^ that has the highest absorbance in the β-phase, 80 °C is the best annealing temperature for the β-phase crystallization of PVDF. However, focusing on the peak (761 cm^−1^) that has the highest absorbance in the α-phase, the annealing temperature does not affect the α-phase crystallization. From the UV-Vis analysis (Figure 6), one can find that there is no special correlation of the absorbance of PVDF to a specific wavelength and the higher concentration of PVDF may raise the absorbance a little bit. From the electrical hysteresis loop (Figure 7), one can find that the case of 15 wt% PVDF shows the highest remnant polarization while those of the other two cases (5 wt% and 10 wt%) are similar. From the C-V curves of Figure 8, one can verify the resulting PVDF films indeed possess ferroelectricity. In summary, the best annealing temperature for the β-phase crystallization of PVDF is 80 °C, which is adopted in the subsequent process of PVDF film GO-doping. Higher PVDF concentrations will benefit remnant polarization but, as mentioned in Section 3.1, this damages the film quality.

### 3.3. The Spin-Coating of Graphene-Oxide-Doped PVDF Film

**Figure 9.**
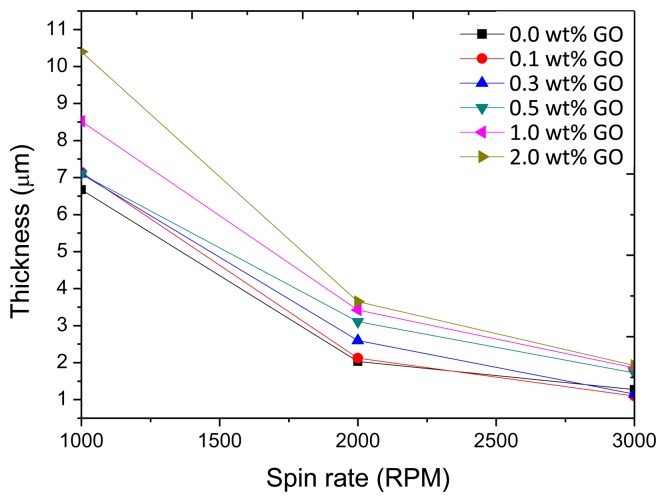
The thickness to spin rate of the GO-doped PVDF film spin-coating.

As shown in Figure 9, the GO doping also affects the spin-coating film thickness, especially for lower spin rates (1,000 rpm), and the higher the GO-doping concentration, the thicker the film.

**Figure 10.**
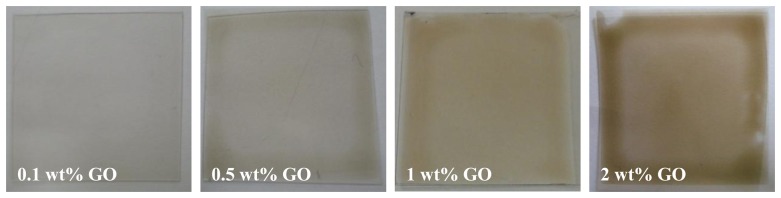
The GO-doped PVDF films with different GO concentrations. The PVDF concentration is 10 wt%.

On the other hand, the film becomes less transparent (Figure 10) as the concentration of GO increases. Furthermore, the film around the border of the substrate is much thicker than that in the central area. The GO-doping will make the substrate warp and, even worse, make the PVDF film peel off.

**Figure 11.**
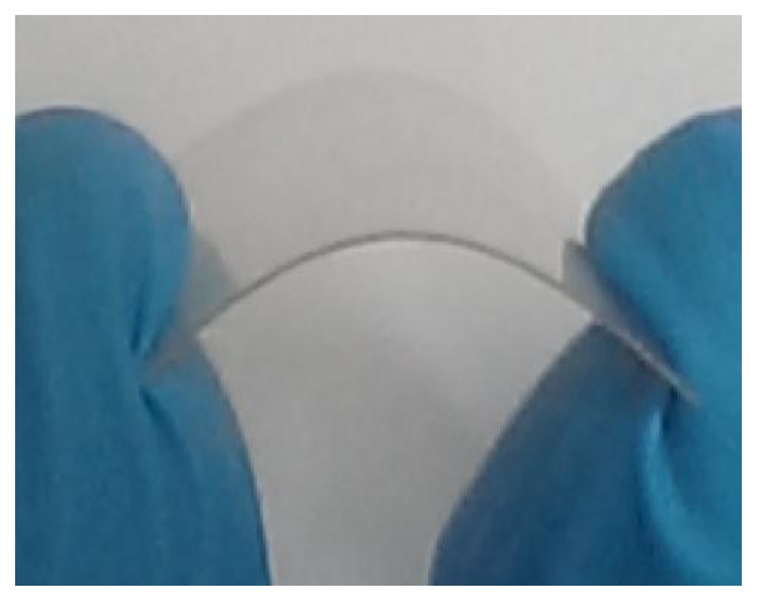
The flexible GO-doped PVDF film.

**Figure 12.**
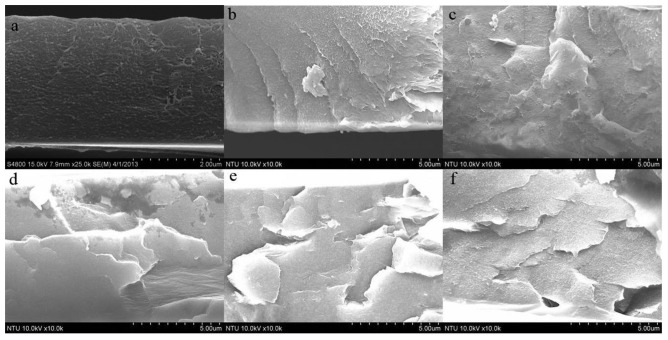
SEM pictures of the cross-sections of PVDF films whose GO-doping concentrations are: (**a**) 0 wt%; (**b**) 0.1 wt%; (**c**) 0.3 wt%; (**d**) 0.5 wt%; (**e**) 1.0 wt%; (**f**) 2.0 wt%, respectively.

The resulting GO-doped PVDF film on ITO/PET substrate is transparent and flexible and thus easily bent with the fingers (Figure 11). Figure 12 shows SEM pictures of the cross sections of PVDF films with different wt% GO-doping. The cross-section of neat PVDF film (Figure 12a), is rather flat and smooth, while those of the other nanocomposite GO-doped PVDF show that most of the GO is fully exfoliated and clearly well dispersed. Furthermore, GO is randomly dispersed within the matrix, which is important for improving the mechanical properties of nanocomposites and increasing the pyroelectricity of PVDF. Consequently, the melt mixing method is appropriate for obtaining a homogeneous distribution of GO nanosheets within the PVDF polymer. In summary, the GO-doping will increase the thickness of film spin-coating, make the film less transparent, warp the substrate, and in the worst case, even make the PVDF film peel off, if the GO-doping concentration is too high. The melt mixing method is appropriate to obtain a homogeneous distribution of GO nanosheets within the PVDF polymer.

### 3.4. The Crystallization of Graphene-Oxide-Doped PVDF

**Figure 13.**
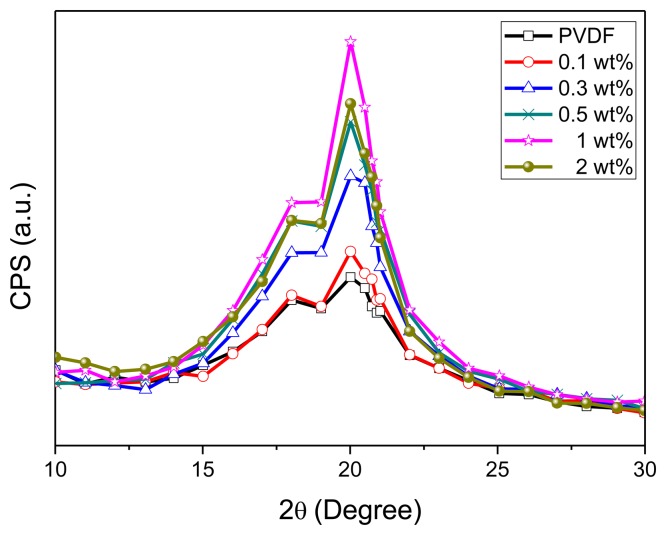
The XRD analysis of 10 wt% PVDF film with different wt% GO doping (0.0, 0.1, 0.3, 0.5, 1.0, and 2.0).

Following the spin-coating process, the annealing process at 80 °C for 2 h is to modify the β-phase crystallization of GO-doped PVDF. From the XRD analysis (Figure 13), one can find the strongest peak at 2θ = 20.8° (the β-phase crystallization) with 1 wt% GO-doping, while the neat PVDF reveals the weakest peak.

**Figure 14.**
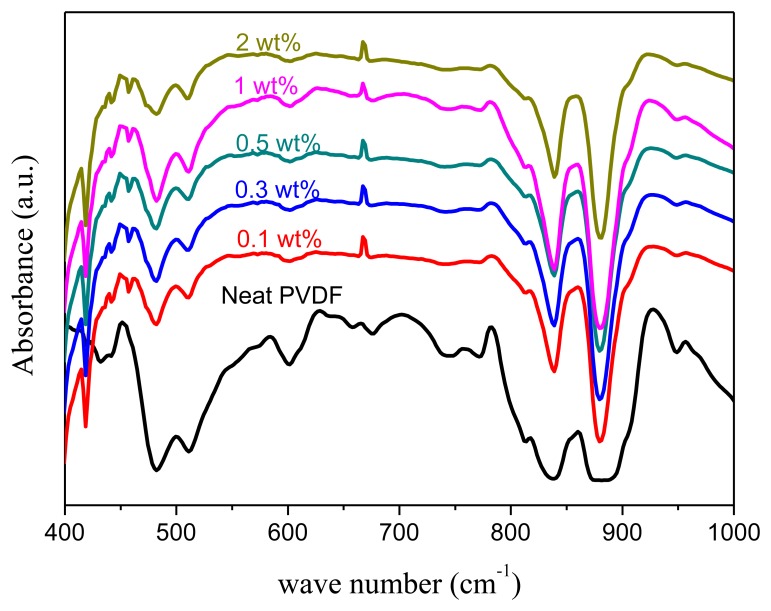
The FTIR analysis of 10 wt% PVDF film with different wt% GO doping (0, 0.1, 0.3, 0.5, 1.0, and 2.0).

The formation of β-phase in PVDF nanocomposite film can be confirmed by the presence of specific transmittance bands in the FTIR spectra. Figure 14 shows the FTIR spectra of GO-doped PVDF. One can find that GO-doping will change the crystallinity of PVDF, but the peak at wave number 839 cm^−1^ still exists and the best doping concentration is 1 wt%, because the wave number at 839 cm^−1^ is the strongest absorbance peak of the β-phase and 1 wt% doping concentration reveals the strongest β-phase peak. The formation of β-phase in PVDF nanocomposites is attributed to the strong and specific interactions between the carbonyl groups found in GO and the CF_2_ segments of the PVDF polymer [21]. In summary, GO-doping significantly benefits the β-phase crystallization of PVDF nanocomposite, and a complete β-phase can be achieved with only 1 wt% GO concentration, which is much less than other literature reports that require high concentrations of CNTs, graphene derivatives, or clay particles. XRD analysis is in good agreement with FTIR analysis.

### 3.5. The Pyroelectricity of Graphene-Oxide-Doped PVDF

**Figure 15.**
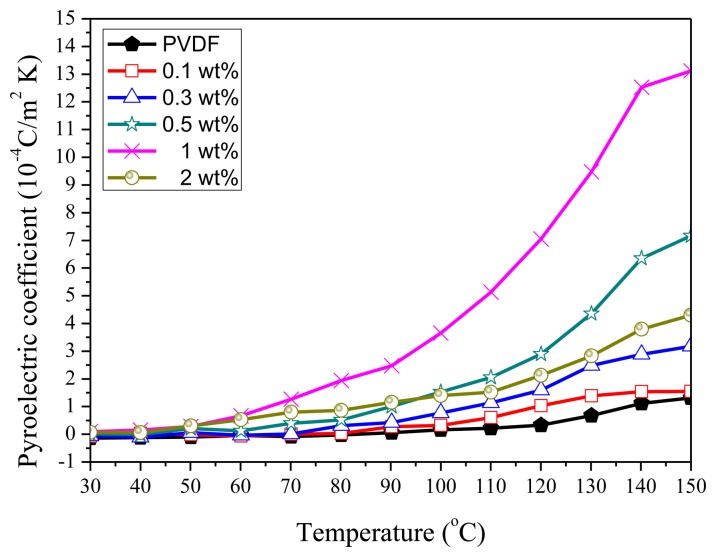
The pyroelectricities of 10 wt% PVDF film with different wt% GO-doping (0.0, 0.1, 0.3, 0.5, 1.0, and 2.0).

**Figure 16.**
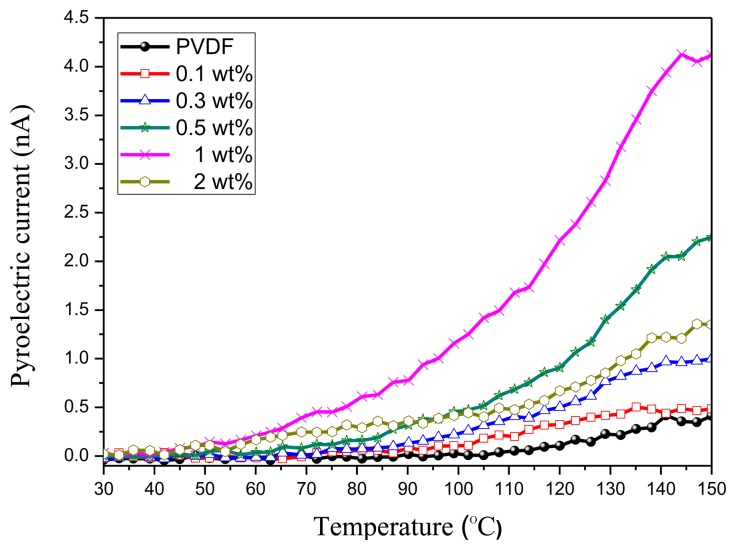
The pyroelectric currents of 10 wt% PVDF film with different wt% GO-doping (0.0, 0.1, 0.3, 0.5, 1.0, and 2.0).

As shown in Figure 15, the largest pyroelectric coefficient occurs when doping 1 wt% GO, a result that coincides with the XRD and FTIR analyses. GO-doping can effectively improve the pyroelectric properties of PVDF. The pyroelectric current (Figure 16) of the case of 1 wt% GO-doping is three times the case of 2 wt% GO-doping and ten times the case without GO-doping. However, too high a GO-doping will cause current leakage. It had been found that high doping concentrations will cause a decreased figure of merit (F_V_) because there will be a significant increase in the dielectric constant and thus the pyroelectric signal decreases.

## 4. Conclusions

This paper presents a methodology for enhancing the pyroelectricity of PVDF by GO doping. The PVDF film with graphene-oxide doping is fabricated by a sol-gel method. A complete study on the process of the graphene oxide doping of PVDF is accomplished. Some key points about the process are addressed by experiments. The main disadvantage of the sol-gel method is the porosity of the resulting films, but this porosity can be effectively eliminated by the use of 10 wt% PVDF solution in DMF and solvent-baking under vacuum. The best annealing temperature for the β-phase crystallization of PVDF is 80 °C when using an ITO/PET substrate. Higher PVDF concentrations will benefit remnant polarization, but will damage the film quality. Graphene-oxide doping will increase the thickness of film spin-coating, make the film less transparent, warp the substrate, and in the worst case, make the PVDF film peel off, if the GO-doping concentration is too high. Graphene-oxide significantly benefits the β-phase crystallization of PVDF nanocomposites, and a complete β-phase can be achieved with only 1 wt% graphene-oxide concentration which is much less than other literature values that require high concentrations of CNTs, graphene derivatives, or clay particles. XRD analysis is in good agreement with FTIR analysis. Graphene-oxide can effectively improve the pyroelectric properties of PVDF, although too much GO-doping will cause current leakage. It had been found that high doping concentrations will cause decreased figure of merit (F_V_) because there will be a significant increase in the dielectric constant and thus the pyroelectric signal will decrease.

## Figures and Tables

**Table 1.** The recipes for GO-doping PVDF.

**Process Steps**	**Issues of Concern**	**Equipment**
PVDF sol preparation	Solvent selectionConcentration of PVDF Concentration of PVDF	Beaker Stirring hot plate Ultrasonic cleaner
Spin-coating of PVDF film	Film uniformity and porosity Solvent baking	Spin coater Stirring hot plate Optical microscope
Annealing of PVDF film	β-phase crystallization of PVDF	XRD FTIR UV-Vis Ferroelectric analyzer
Graphene oxide doping of PVDF	Concentration of graphene oxide	Beaker Stirring hot plate Ultrasonic cleaner SEM
Annealing of PVDF film with graphene oxide doping	β-Phase crystallization of PVDF	XRD FTIR
Pyroelectricity measurement of GO-doped PVDF Film	Temperature variation rate control Tiny pyroelectric current measurement	Thermoelectric cooler Current-to-voltage converter Multimeter

**Table 2.** The PVDF GO-doping recipes.

**Recipe**	**GO**	**PVDF**	**DMF**
0	0 mg (0.0 wt%)	1 g (10 wt%)	9 g
1	1 mg (0.1 wt%)		
2	3 mg (0.3 wt%)		
3	5 mg (0.5 wt%)		
4	10 mg (1.0 wt%)		
5	20 mg (2.0 wt%)		
